# Supplementary material for: Paranoia, sensitization and social inference: findings from two large-scale, multi-round behavioural experiments
Source: R Soc Open Sci. 2020 Mar 11;7(3):191525. doi: 10.1098/rsos.191525 (PMC7137981; doi:10.1098/rsos.191525)
Supplement: Supplementary Materials [file rsos191525supp1.docx]

**Supplementary Material**

**Paranoia, sensitisation, and social inference: findings from two large-scale, multi-round behavioural experiments**

Barnby, J.M., Deeley, Q., Robinson, O., Raihani, N., Bell, V., Mehta., M.A.

*Appendix A* **–** The multi-round dictator game task schematic for one partner.

**
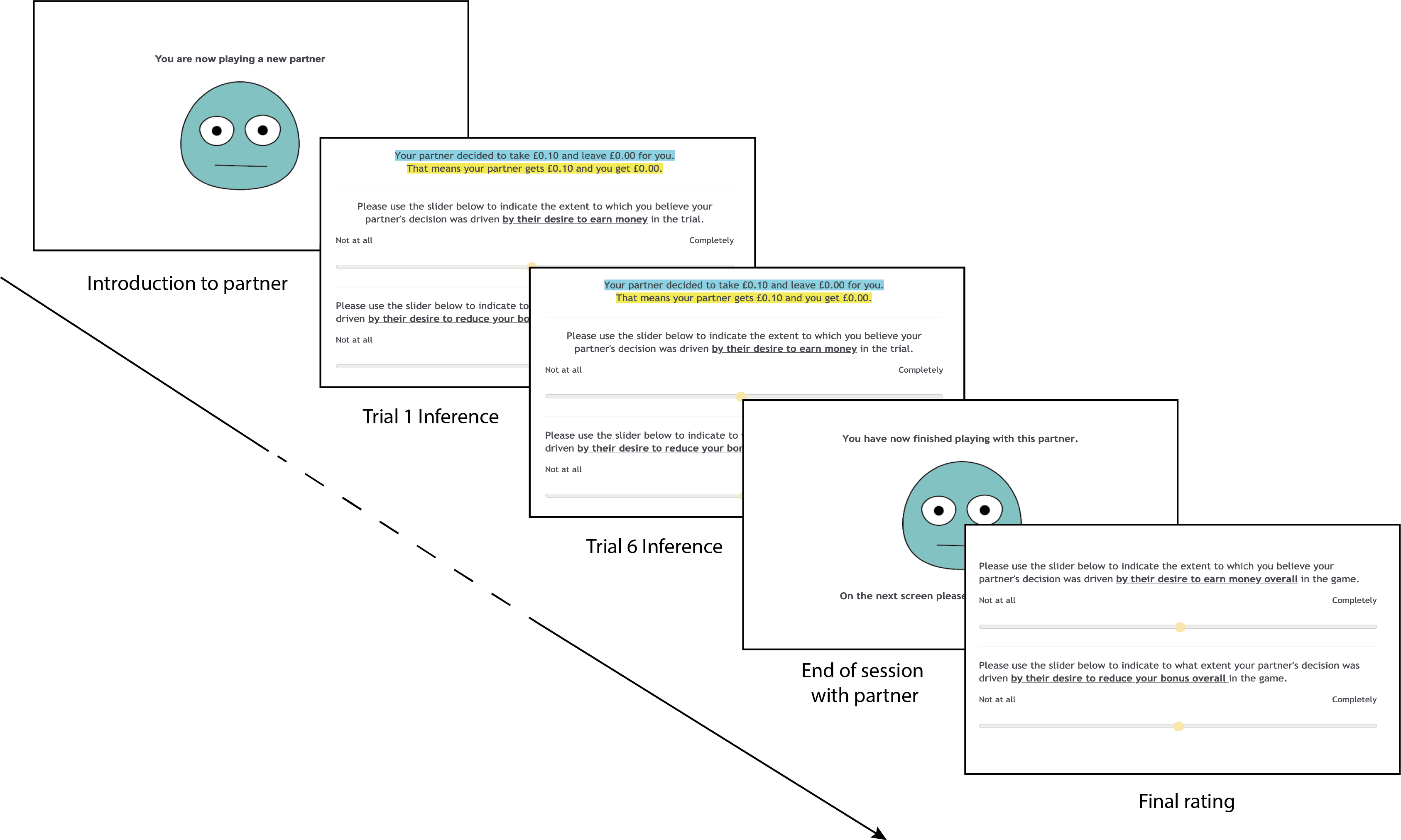
**

*Appendix B* **–** Trials-to-peak-decision for Harmful intent and Self-Interest scores

**Variables affecting earlier trial-to-peak-decision for Harmful Intent attributions within unfair and fair dictator decisions in the multi-round dictator game (Study 1).** Trials where 53.51 (unfair) or 24.26 (fair) was first triggered were coded like so: 6 < 5 < 4 < 3 < 2 < 1 < 0, where 0 means over the mean was never scored or an earlier trial scored over the mean. Relative Importance is the probability that the term in question is a component of the true best model.

| Parameter | Estimate | Standard  Error | 95% CI | | Relative Importance |
| --- | --- | --- | --- | --- | --- |
|  |  |  | Lower | Upper |  |
| **Unfair Dictator** | | | | | |
| *Intercept 6\|5* | -2.49 | 0.09 | -2.66 | -2.32 |  |
| *Intercept 5\|4* | -2.40 | 0.08 | -2.57 | -2.24 |  |
| *Intercept 4\|3* | -2.37 | 0.08 | -2.54 | -2.21 |  |
| *Intercept 3\|2* | -2.33 | 0.08 | -2.50 | -2.17 |  |
| *Intercept 2\|1* | -2.30 | 0.08 | -2.47 | -2.14 |  |
| *Intercept 1\|0* | -2.28 | 0.08 | -2.44 | -2.11 |  |
| **Paranoia** (Z score) | -0.12 | 0.05 | -0.21 | -0.02 | 1 |
| **Age** | -0.01 | 0.02 | -0.06 | 0.02 | 0.32 |
| **Sex** (Male \| Female) | 0.01 | 0.05 | -0.14 | 0.26 | 0.21 |
| **Fair Dictator** | | | | | |
| *Intercept 6\|5* | -2.61 | 0.10 | -2.81 | -2.40 |  |
| *Intercept 5\|4* | -2.51 | 0.10 | -2.71 | -2.31 |  |
| *Intercept 4\|3* | -2.47 | 0.10 | -2.66 | -2.27 |  |
| *Intercept 3\|2* | -2.43 | 0.10 | -2.63 | -2.23 |  |
| *Intercept 2\|1* | -2.39 | 0.10 | -2.59 | -2.20 |  |
| *Intercept 1\|0* | -2.38 | 0.10 | -2.58 | -2.18 |  |
| **Age** | -0.02 | 0.02 | -0.07 | 0.01 | 0.69 |
| **Paranoia** (Z score) | -0.06 | 0.06 | -0.19 | 0.01 | 0.55 |
| **Sex** (Male \| Female) | 0.01 | 0.04 | -0.15 | 0.26 | 0.13 |

**Variables affecting earlier trial-to-peak-decision for Self-Interest within unfair dictator decisions in a multi-round dictator game (Study 1).** Trials where 60 was triggered were coded like so: 6 < 5 < 4 < 3 < 2 < 1 < 0, where 0 means 60 was never scored or an earlier trial scored 60. Relative Importance is the probability that the term in question is a component of the true best model.

| Parameter | Estimate | Standard  Error | 95% CI | | Relative Importance |
| --- | --- | --- | --- | --- | --- |
|  |  |  | Lower | Upper |  |
| **Intercept 6\|5** | -1.64 | 0.04 | -1.72 | -1.57 |  |
| **Intercept 5\|4** | -1.63 | 0.04 | -1.70 | -1.55 |  |
| **Intercept 4\|3** | -1.63 | 0.04 | -1.70 | -1.55 |  |
| **Intercept 3\|0** | -1.63 | 0.04 | -1.70 | -1.55 |  |
| **Paranoia** (Z score) | 0.01 | 0.08 | -0.14 | 0.17 | 0.21 |
| **Age** | 0.01 | 0.08 | -0.15 | 0.16 | 0.21 |

**Variables affecting earlier trial-to-peak-decision for Self-Interest within fair dictator decisions in a multi-round dictator game (Study 1).** Trials where 60 was triggered were coded like so: 6 < 5 < 4 < 3 < 2 < 1 < 0, where 0 means 60 was never scored or an earlier trial scored 60. Relative Importance is the probability that the term in question is a component of the true best model.

| Parameter | Estimate | Standard  Error | 95% CI | | Relative Importance |
| --- | --- | --- | --- | --- | --- |
|  |  |  | Lower | Upper |  |
| **Intercept 6\|5** | -2.37 | 0.05 | -2.47 | -2.26 |  |
| **Intercept 5\|4** | -2.27 | 0.05 | -2.38 | -2.17 |  |
| **Intercept 4\|3** | -2.23 | 0.05 | -2.33 | -2.13 |  |
| **Intercept 3\|2** | -2.20 | 0.05 | -2.30 | -2.10 |  |
| **Intercept 2\|1** | -2.18 | 0.05 | -2.28 | -2.08 |  |
| **Intercept 1\|0** | -2.17 | 0.05 | -2.26 | -2.07 |  |
| **Age** | 0.04 | 0.10 | -0.15 | 0.23 | 0.22 |
| **Sex** (Male \| Female) | 0.03 | 0.09 | -0.16 | 0.22 | 0.22 |

*Appendix C – Correlation coefficients of all baseline variables.* Top panel: Age, GPTS, STAI-T, IPS (total measure), and PSWQ. Bottom panel: GPTS, Interpersonal awareness, Need for Attachment, Separation Anxiety, Timidity, and Fragile Inner Self Subscale of the IPS.


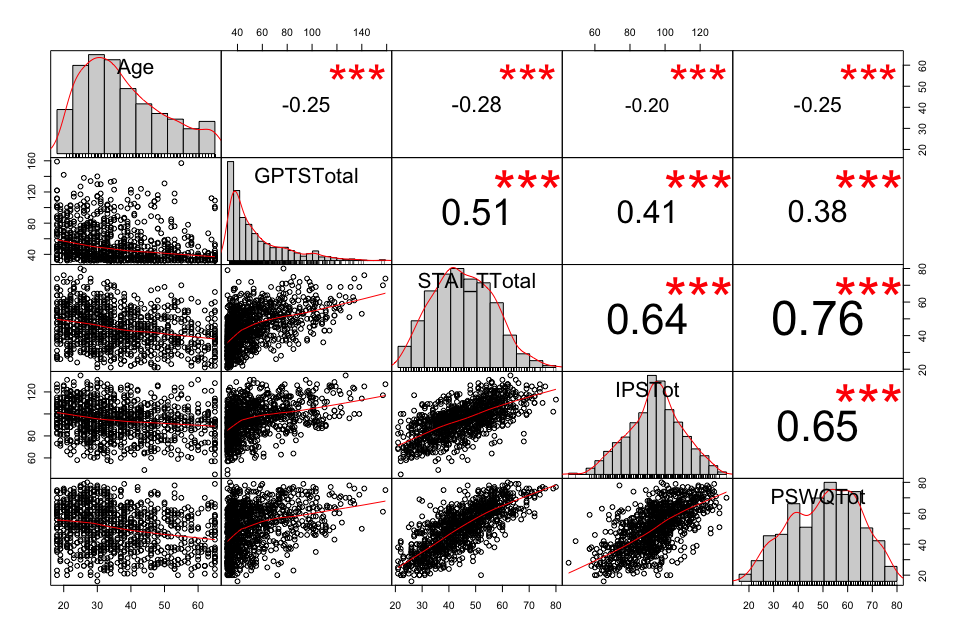

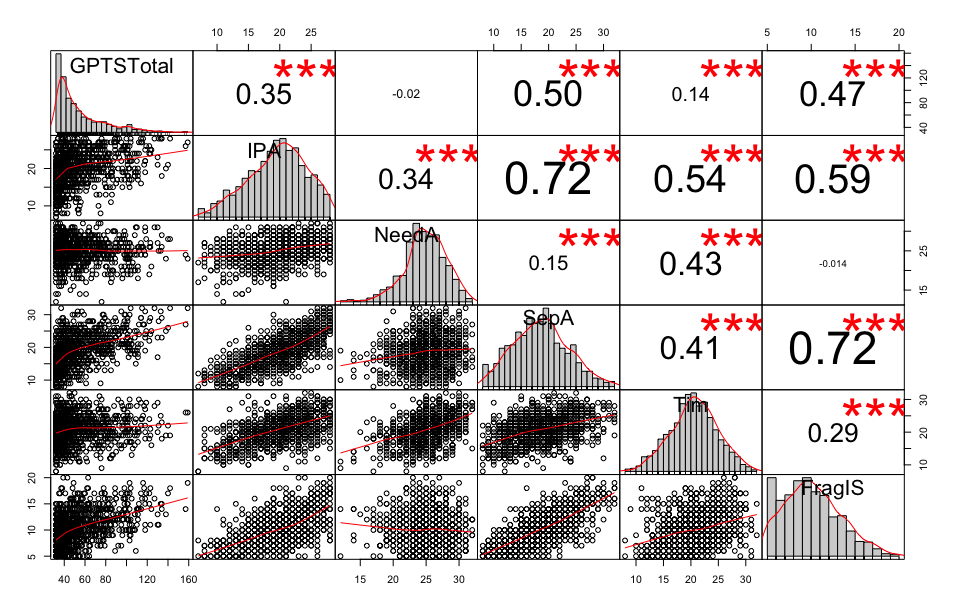


*Appendix D –* **Summary of extra explanatory variables affecting Harmful Intention and Self Interest attributions in the multi-round dictator game including paranoia in all models (Study 2).** Harmful Intent was coded as a five-level ordinal categorical variable and set as the response term in the clmm. ID was set as the random variable (42). Relative Importance is the probability that the term in question is a component of the true best model. Numbers denote the model that the parameter belonged to for each outcome variable. E.g. under Harmful intent Attributions, Separation Anxiety and Timidity were run in the same model (4) but separate to Trait Anxiety (1). NA = not included in the final top model.

| Model | Parameter | Estimate | Standard  Error | 95% CI | | Relative Importance |
| --- | --- | --- | --- | --- | --- | --- |
|  |  |  |  | Lower | Upper |  |
|  | **Harmful Intent Attributions** | | | | | |
| 1 | Trait Anxiety | -0.19 | 0.12 | -0.42 | 0.05 | 1 |
| 2 | State Anxiety | 0.00 | 0.04 | -0.19 | 0.23 | 0.14 |
| 3 | Interpersonal Sensitivity | -0.29 | 0.04 | -0.49 | -0.10 | 1 |
| 4 | Interpersonal Awareness | -0.54 | 0.13 | -0.80 | -0.28 | 1 |
| 4 | Separation Anxiety | 0.36 | 0.14 | 0.08 | 0.64 | 1 |
| 4 | Timidity | -0.02 | 0.07 | -0.34 | 0.14 | 0.22 |
| 4 | Need for Attachment | -0.01 | 0.03 | -0.09 | 0.07 | 0.19 |
| 4 | Fragile Inner Self | 0.01 | 0.07 | -0.22 | 0.38 | 0.18 |
| 5 | Worry | -0.06 | 0.06 | -0.18 | 0.05 | 1 |
| 1-5 | Paranoia (Range) | 0.34 -  0.60 | 0.06 -  0.14 | 0.13 – 0.38 | 0.54 -  0.88 | 1-1 |
|  | **Self Interest Attributions** | | | | | |
| 6 | Trait Anxiety | 0.05 | 0.08 | -0.06 | 0.29 | 0.41 |
| 7 | State Anxiety | 0.14 | 0.12 | -0.01 | 0.38 | 0.76 |
| 8 | Interpersonal Sensitivity | NA | NA | NA | NA | NA |
| 9 | Interpersonal Awareness | 0.31 | 0.14 | 0.04 | 0.58 | 1 |
| 9 | Separation Anxiety | -0.02 | 0.07 | -0.38 | 0.15 | 0.16 |
| 9 | Timidity | -0.46 | 0.11 | -0.68 | -0.23 | 1 |
| 9 | Need for Attachment | 0.28 | 0.11 | 0.07 | 0.48 | 1 |
| 9 | Fragile Inner Self | -0.05 | 0.10 | -0.38 | 0.10 | 0.35 |
| 10 | Worry | 0.11 | 0.11 | -0.11 | 0.32 | 0.66 |
| 6-10 | Paranoia (Range) | -0.03 -  -0.10 | 0.05 -  0.11 | -0.28 –  -0.35 | 0.02 – 0.11 | 0.31 –  0.58 |


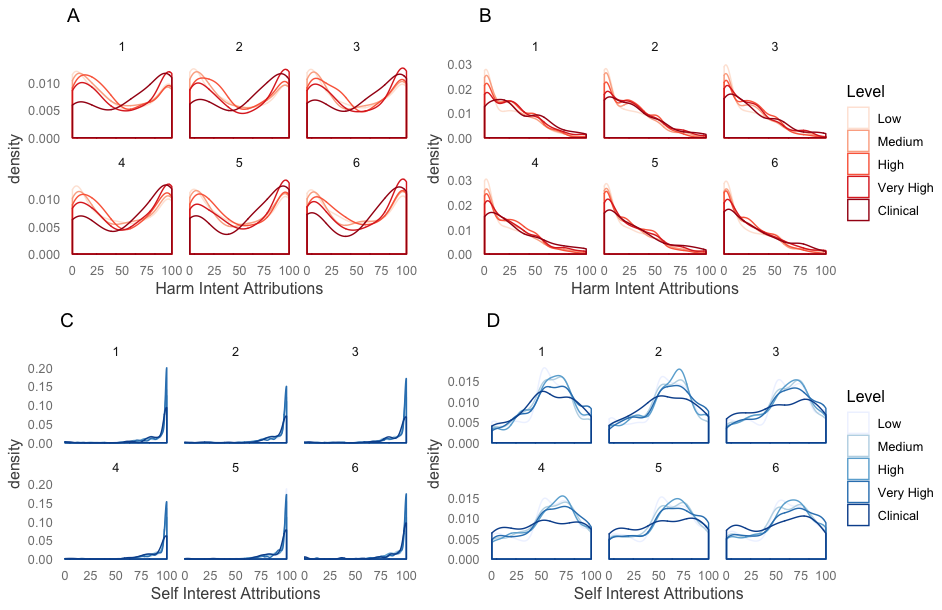
*Appendix E –* Density distributions for attributions. **A & B:** Density distributions for Harmful Intent scores within each trial (1-6) for unfair (A) and fair (B) dictators for each level of paranoia. **C & D:** Density distributions for Self Interest scores within each trial (1-6) for unfair (C) and fair (D) dictators for each level of paranoia.

*Appendix F –* Beta coefficient 95% confidence intervals from linear mixed effects models for harmful intent and self-interest attributions for paranoia, dictator, and sex.

|  | **Harmful Intent Attributions** | | | | **Self Interest Attributions** | | | |
| --- | --- | --- | --- | --- | --- | --- | --- | --- |
|  | **Unfair Dictator** | | **Fair Dictator** | | **Unfair Dictator** | | **Fair Dictator** | |
| 95% CI | *2.50%* | *97.50%* | *2.50%* | *97.50%* | *2.50%* | *97.50%* | *2.50%* | *97.50%* |
| .sig01 | 34.85 | 37.27 | 20.73 | 22.19 | 11.03 | 11.83 | 25.76 | 27.57 |
| .sigma | 11.09 | 11.42 | 9.04 | 9.31 | 6.93 | 7.14 | 9.52 | 9.81 |
| (Intercept) | 46.66 | 51.04 | 23.31 | 25.99 | 92.29 | 93.80 | 56.23 | 59.51 |
| zPara | 1.93 | 5.34 | 0.91 | 2.96 | -0.74 | 0.36 | -1.29 | 1.24 |
| Trial2 | -0.13 | 1.36 | -1.21 | 0.00 | -0.25 | 0.68 | -1.21 | 0.06 |
| Trial3 | 0.62 | 2.11 | -2.07 | -0.86 | -0.21 | 0.72 | -0.89 | 0.39 |
| Trial4 | 1.46 | 2.95 | -2.19 | -0.98 | -0.23 | 0.70 | -1.25 | 0.03 |
| Trial5 | 2.16 | 3.65 | -2.23 | -1.02 | 0.08 | 1.01 | -1.20 | 0.08 |
| Trial6 | 2.75 | 4.24 | -2.38 | -1.16 | -0.46 | 0.47 | -1.60 | -0.33 |
| SexMale | -5.22 | 1.85 | -4.03 | 0.20 | -1.23 | 1.06 | -3.29 | 1.95 |
